# Radiotherapy-induced neurocognitive decline among adult intracranial tumor patients: A voxel-based approach

**DOI:** 10.1093/neuonc/noaf114

**Published:** 2025-05-04

**Authors:** Charlotte Sleurs, Catharina M L Zegers, Marvin F Ribeiro, Wouter van Elmpt, Jeanette Dijkstra, Alida A Postma, Laurien De Roeck, Karin Gehring, Wouter De Baene, Margriet M Sitskoorn, Maarten Lambrecht, Daniëlle B P Eekers

**Affiliations:** Department of Oncology, KU Leuven, Leuven, Belgium; Department of Cognitive Neuropsychology, Tilburg University, Tilburg, The Netherlands; Department of Radiation Oncology (Maastro), GROW Research Institute for Oncology and Reproduction, Maastricht University Medical Centre+, Maastricht, The Netherlands; Department of Radiology & Nuclear Medicine, Maastricht University Medical Center+, Mental Health and Neuroscience research institute, Maastricht, The Netherlands; Department of Radiation Oncology (Maastro), GROW Research Institute for Oncology and Reproduction, Maastricht University Medical Centre+, Maastricht, The Netherlands; Department of Radiation Oncology (Maastro), GROW Research Institute for Oncology and Reproduction, Maastricht University Medical Centre+, Maastricht, The Netherlands; Department of Medical Psychology, Maastricht University Medical Centre+, Maastricht, The Netherlands; Department of Radiology & Nuclear Medicine, Maastricht University Medical Center+, Mental Health and Neuroscience research institute, Maastricht, The Netherlands; Department of Radiation Oncology, UZ Leuven, Leuven, Belgium; Department of Neurosurgery, Elisabeth-TweeSteden Hospital, Tilburg, The Netherlands; Department of Cognitive Neuropsychology, Tilburg University, Tilburg, The Netherlands; Department of Cognitive Neuropsychology, Tilburg University, Tilburg, The Netherlands; Department of Cognitive Neuropsychology, Tilburg University, Tilburg, The Netherlands; Department of Radiation Oncology, UZ Leuven, Leuven, Belgium; Department of Radiation Oncology (Maastro), GROW Research Institute for Oncology and Reproduction, Maastricht University Medical Centre+, Maastricht, The Netherlands

**Keywords:** cranial radiation, intracranial tumors, neurocognitive sequelae, voxel-based image analysis

## Abstract

**Background:**

Cranial irradiation is a key component of neuro-oncological treatment but can result in cognitive side effects. Preserving cognition from radiotherapy-(RT)-induced toxicity remains an ongoing debate. To spatially map radiotoxic effects in patients who underwent cranial RT, this study applied a voxel-based approach.

**Methods:**

Cognitive assessments (Controlled Word Association (COWA), Hopkins Verbal Learning (HVLT-R), and Trail Making Tests (TMT A,B)) were conducted prospectively before, 6 months and 1 year post-RT in 111 intracranial tumor patients (18–80 years). Reliable change indices indicated cognitive changes across timepoints. CT and T1-weighted MRI scans acquired at diagnosis were co-registered, normalized to standard space, and smoothed. Voxel-wise permutation-based regression analyses examined the relationship between RT dose and cognitive decline (α < 0.05 at cluster level).

**Results:**

Images of 111 patients (*Mdn* age = 55.39 years; 47% male; lesions were gliomas (61%), meningiomas (18%), other (21%); in frontal (33%), temporal (25%), other location (42%)) were analyzed. Reliable decline was most pronounced at 6 months, particularly on the TMT A (25.77%), TMT B (24.21%), and HVLT immediate recall (21%). At 1 year, 20% of patients continued to show a decline in TMT B. Higher RT doses to frontal gyri, temporal, occipital, and para-central regions were associated with declines in verbal fluency, memory, processing speed, and flexibility at both peak- and cluster-level.

**Conclusion:**

Differential voxel-wise RT dose effects at peak versus cluster level suggest local and network-based recruitment of diverse functional regions and vulnerability to cranial RT. These insights may help re-define key regions at risk from a network-based perspective, preserving cognition in future RT planning.

**Key Points**

- Decline was most pronounced shortly post-RT in processing speed, flexibility, and memory.
- RT doses to frontal, temporal, and occipital regions were linked to cognitive decline.
- Differential voxel-wise significance suggests local and network-based RT effects.

**Importance of the Study**

This study highlights the impact of cranial radiotherapy (RT) on cognitive function in patients with intracranial tumors. Reliable decline in cognitive performance was most pronounced at 6 months post-RT, with about 20% of patients still affected at 1 year in cognitive flexibility. By using a voxel-based analysis, we found that higher RT doses to specific brain regions—frontal, temporal, occipital, and para-central areas—were linked to a decline in specific cognitive outcomes, particularly in verbal fluency, memory, processing speed, and cognitive flexibility. By identifying focal as well as cluster regions at risk, these voxel-wise findings suggest local as well as network-based recruitment of diverse functional regions. This underscores the importance of adopting a network-based approach in radiation planning, to safeguard functional key brain regions, which may lead to improved quality of life for cancer patients undergoing RT.

Radiotherapy (RT) is a cornerstone in the oncological treatment of intracranial tumors.^1^ While the clinical significance of cranial RT in improving survival rates is well-established, better understanding of its potential side effects is needed.^2^ Cranial RT is known to directly as well as indirectly induce neural damage and to potentially lead to extended molecular and cellular neurotoxic mechanisms, including decreased neurogenesis, altered neurotransmission, inflammatory responses, demyelination, and atrophy.^3–5^ Potential sequelae in daily life that are directly related to such neurotoxicity, are neurobehavioral and cognitive symptoms, including a decline in attention, memory, or executive functioning.^6–8^ These symptoms can largely affect daily life functioning, participation in society, and quality of life.^9–11^ As survival rates of intracranial tumors have increased throughout the last decades, there is a growing focus on such potential cognitive sequelae of the oncological treatment, including cranial irradiation.^8,12,13^ Advanced neuroimaging modalities have emerged as crucial tools to assist in unraveling detrimental effects of cranial RT to brain tissue.^14–16^ Such techniques, including multiple magnetic resonance imaging (MRI) sequences (eg, diffusion-weighted MRI, functional MRI) and metabolic imaging (eg, positron emission tomography) have provided crucial insights into the structural and functional brain alterations induced by radiation,^17–24^ aiding in the identification of both underlying neurotoxic mechanisms as well as location-specific effects. An increasing number of studies explored associations between these brain changes and observed cognitive deficits in brain tumor patients, showing alterations in gray and white matter volumes,^25,26^ in structural and functional connectivity and in the brain network organization^27–30^ due to RT, each being associated with diverse cognitive outcomes.^31,32^ Precise and function-informed radiation planning is key to balance the effective tumor control with minimal damage to these OARs, limiting the potential radiotoxicity and debilitating side effects. Therefore, recently developed clinical guidelines in radiation oncology defined potential key intracranial OARs relevant for cognition, such as the hippocampus, prefrontal cortex, cerebellum, basal ganglia, and thalamus.^33–35^ However, it remains to be determined whether these anatomically delineated OARs effectively represent the key *functional regions* in the brain for daily life functioning. Consequently, the selection and prioritization of OARs to spare from toxicity, with the aim of preserving cognition, is still widely debated. By elucidating the spatial and functional aspects of radiation-induced changes, clinicians can refine treatment strategies, minimizing the potential cognitive sequelae, and ultimately improving the overall quality of life for individuals with brain tumors. A comprehensive understanding of causal relationships between localized radiation doses to the brain and daily life cognitive functioning is crucial, to elucidate location-specific neurotoxic mechanisms and, consequently, predicting cognitive decline in a more targeted manner. This study therefore aims to map the spatially distributed radiotoxic effects with a clinical impact on cognitive changes throughout therapy. From a neuroscientific perspective, a voxel-based approach is applied to the 3D-delivered cranial radiation dose distribution to predict changes in cognitive functioning. This approach allows us to determine the eloquent brain areas that could be important *regions at risk* that need to be spared from (high-dose) cranial RT.

## Methods

### Patients

Patients with intracranial tumors, aged 18 to 80, who received photon (and proton) beam cranial radiation between April 2019 and July 2022, were included in the study. Histological tumor subtypes included meningiomas, gliomas, vestibular schwannomas, pituitary adenomas, craniopharyngiomas, one chondrosarcoma, paraganglioma, and chordoma. Individuals were excluded if they were unable to perform the tests due to malaise, substantial hearing or vision loss, chronic fatigue, or those with baseline computerized tomography (CT) or magnetic resonance imaging (MRI) scans of insufficient quality. Finally, patients who received re-irradiation to the brain during the follow-up period were excluded from the analysis. All participants signed an informed consent for their agreement on the scientific use of the clinical, imaging, and demographical data. This study was approved by the medical ethics committee of the University Hospital of Maastricht (MUMC).

### Materials

Before the start of RT treatment, each patient first underwent one high-resolution MRI scan on a 3 Tesla MRI scanner (Ingenia CX or Achieva Philips system). This MR imaging protocol included an axial T1-weighted sequence (T1w) with 1 mm slice thickness pre-contrast, which was enhanced with gadolinium for post-contrast acquisition (Gadovist© 1.0 mmol/ml at 0.1 mL/kg body weight). For each patient a planning CT-scan was performed, which was fused with the T1-weighted MRI scan (to delineate the OARs according to the European Particle Therapy Network guideline^35^) and for radiotherapy treatment planning. Both a photon and proton beam radiation plan was then simulated for dose comparison, based on which patients with a dosimetric advantage of protons over photons, were treated with proton beam radiotherapy. Besides imaging acquisition, all patients prospectively underwent cognitive follow-up as an integral part of their standard care. Compliance rates for the neurocognitive assessments were high across timepoints (ie, 75.3%–90.4%), with data missing mostly due to missed appointments or illness. Detailed information on compliance was published previously.^36^ Specifically, a cognitive assessment was administered prior to the initiation of radiation treatment, at 6 months and at 1-year follow-up. This evaluation involved the Controlled Oral Word-Association Test (COWA),^37^ Hopkins Verbal Learning Test Revised (HVLT-R),^38^ and the Trail Making Test,^39^ with a total duration of approximately 30 min. For each cognitive test score (COWA phonemic fluency, HVLT-R immediate recall, HVLT-R delayed recall, TMT A reaction time, TMT B reaction time), reliable change indices (RCIs) were calculated to estimate the change in cognitive performance between the follow-up timepoint and baseline (ie, in phonemic fluency, immediate and delayed recall, processing speed, and cognitive flexibility, respectively). By using the RCI formula: ((patient score at T2 − patient score at T1) − (mean control group at T2 − mean control group at T1))/(standard error of the difference), practice effects were taken into account.^40^

### Preprocessing of Scans and Delivered Radiation Dose Distribution Images

In preparation for the voxel-wise analyses, gross tumor volumes and physical radiation dose maps were co-registered to anatomical MRI and CT images and spatially normalized to the standard MNI-ICBM152 brain template. Physical dose maps were used, representing the effectively delivered physical doses. Dose maps were smoothed, and voxels containing tumoral tissue were excluded from the analyses. For a detailed description of these procedures, see Supplementary Materials.

### Statistical Analyses

First, descriptive statistics were analyzed for RCI scores from baseline to 6m and to 1y follow-up timepoints (ie, means and standard deviations), and the prevalence of a reliable change with a 95% confidence interval was reported (ie, of RCI < −1.96).

Second, Pearson correlations were calculated for interdependencies between RCIs, and classified as weak, moderate, strong: −0.01 ≤*r*≤ 0.3, 0.3 < *r*≤ 0.5, 0.5 < *r*, respectively.^41^ For the visual presentation of these values, correlation matrices were created.

Finally, voxel-wise statistical analyses were conducted on radiation dose images, to analyze the spatial dose distributions’ impact cognitive decline (ie, RCI scores) in the short- (6 months) and longer run (1 years), using the Statistical nonParametric Mapping toolbox. A permutation-based linear regression model was tested with 5000 permutations. An implicit mask excluding voxels with value 0 was applied. Resulting voxel-based statistics were interpreted both according to significance at peak and at the cluster level (see Suppl. Materials for their definitions). Clusters at both uncorrected and at Family-Wise Error (FWE-) corrected levels were presented.

## Results

From the initial database of 179 subjects with qualitative anatomical scans and GTVs available,^42^ 149 patients had non-recurrent cancer. After image and cognitive assessment quality checks, 2 patients were removed from the dataset due to distorted radiation dose maps or CT images, and 36 patients due to missing data at follow-up, resulting in a dataset of 111 patients for the final analyses. Descriptive patient characteristics are demonstrated in Table 1. The most common tumor location was the frontal lobe (ie, 33.33%), and the most common histology a glioma subtype (ie, 61.26%). Most patients underwent surgery (ie, 62.16%). Both sexes as well as hemispheric tumor locations were relatively equally distributed (Table 1).]

**Table 1.** Characteristics of Included Patient Population

Characteristics	*N* (*n* = 111) patients		Percentage	
**Demographic**				
Median age (SD)	55.39 (14.32)			
Gender: Females	59		53.15%	
**Tumor location**				
(Supra-)sellar region	11		9.91%	
Cerebellar region	17		15.32%	
Frontal(-parietal) region	37 (1)		33.33% (0.90%)	
Occipital region	1		0.90%	
Parietal(-occipital) region	12 (4)		10.81% (3.60%)	
Temporal region	28		25.23%	
**RT specifications**				
Median Dmax (SD)	56.72 (6.35) Gy			
Range Dmax	42.74-76.44 Gy			
Proton beam radiation*	47		42%	
Photon beam radiation	64		58%	
**Involved hemisphere**				
Left-sided	49		44.14%	
Right-sided	51		45.95%	
Bilateral	11		9.91%	
**Surgery**				
No surgery	27		24.32%	
Biopsy	15		13.51%	
Resection	69		62.16%	
**Tumor histology**				
Gliomas	68		61.26%	
Meningiomas	20		18.02%	
Vestibular schwannomas	9		8.12%	
Pituitary adenomas	8		7.21%	
Craniopharyngiomas	3		2.70%	
Other*	3		2.70%	
WHO grade* 1 2 3 4	Gliomas 2/68 29/68 14/68 23/68	Meningiomas 9/20 9/20 2/20 NA	Gliomas 2.94% 42.65% 20.59% 33.82%	Meningiomas 45.00% 45.00% 10.00% NA
**Cognitive assessment**	Mdn (IQR) RCI BL-6m	Mdn (IQR) RCI BL-1y	RCI < -1.96 BL-6m	RCI < -1.96 BL-1y
COWA	−0.79 (−1.47;0.16)	0.03 (−0.79; 0.98)	17.17%	5.41%
HVLT-R immediate	−0.49 (−1.55;0.58)	−0.03 (−1.25; −0.81)	21.00%	9.46%
HVLT-R delayed	−0.36 (−1.58; −0.85)	−0.36 (−0.97; −0.85)	19.39%	15.07%
TMT A	−0.37 (−2.00; 0.71)	−0.01 (−0.92; 1.43)	25.77%	10.96%
TMT B	−1.27 (−1.80; −0.41)	−1.27 (−1.73; −0.51)	24.21%	19.72%

*Note.* *For all patients who received proton beam irradiation, combined treatment was implemented including both proton and photon beam irradiation. * Other category includes one chondrosarcoma, paraganglioma, and chordoma. *WHO grades are shown for gliomas and meningiomas, while all other subtypes were classified as WHO grade 1. The WHO grade was assigned at the moment of diagnosis, according to the contemporaneous criteria. IQR = Interquartile Range. Mdn = Median. NA = Not applicable (meningiomas maximally reach WHO grade 3). N = number. RCI = Reliable Change index. SD = Standard Deviation.

**Figure 1.**
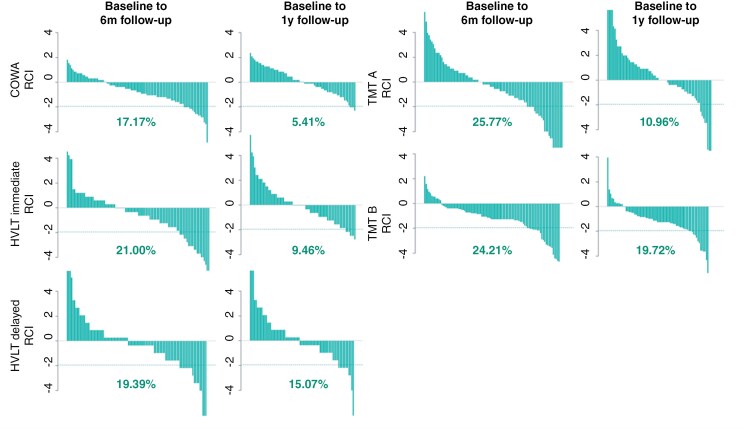
Waterfall plots with Reliable Change Index (RCI) distributions *Note.* This figure presents waterfall plots, showing the distributions of Reliable Change Index (RCI) scores that were calculated from baseline to 6 month (6m) and from baseline to 1 year (1y) follow-up. This was performed for each of the tests, including the Controlled Oral word association (COWA), the Hopkins Verbal learning test (HVLT-R) and Trail Making Test A and B (TMT A and B). A cut-off line is presented at RCI = -1.96, indicating a reliable change with a 95% confidence interval. Percentages of patients reaching this cut-off are depicted below each waterfall plot.

Regarding changes in neuropsychological performance, all median RCI values were within one standard deviation of zero, except for the TMT B, of which the median change score reached −1.06 at 6-month follow-up, and −1.13 at 1-year follow-up. RCI distributions across participants are displayed in Figure 1 (baseline z-scores are depicted in Supplementary Figure 1). Prevalence rates of patients at 6-month follow-up suggest that about 1 out of 4 patients demonstrated a reliable cognitive decline in TMT A and B scores, approximately 1 out of 5 patients in both HVLT-R scores, and 1 out of 6 patients in COWA scores. Even though all RCI scores improved from baseline to 1-year follow-up (compared to earlier follow-up), still about 1 out of 5 patients demonstrated a reliable decline in TMT B scores, which was most common, followed by 1 out of 6 patients with a decline in HVLT-R delayed recall (and 1 out of 10 to 20 for the remaining tests). Cognitive decline was therefore most frequently observed in the TMT tests, with the TMT B remaining most sensitive at 1-year follow-up.

**Figure 2.**
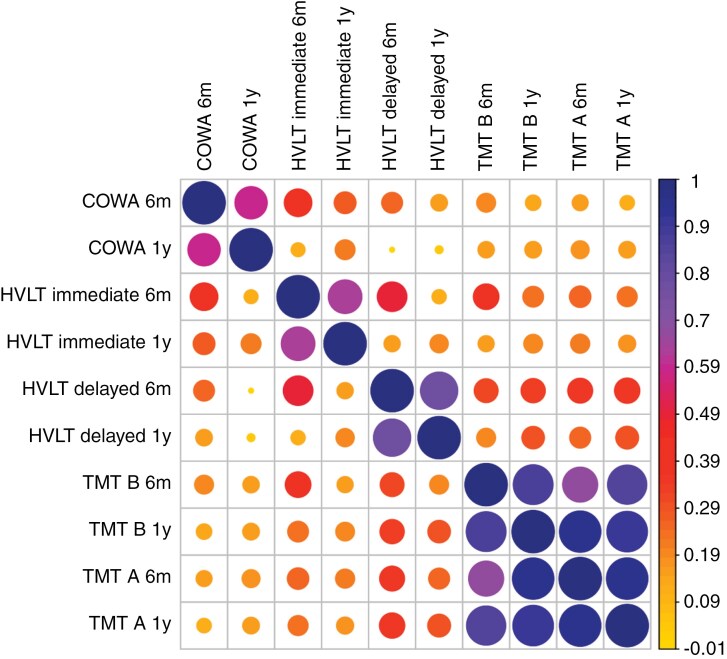
Correlation matrix presenting associations between the RCIs of the different subtests *Note*. Correlation values are depicted as colored circles, where larger circles indicate higher values. The correlation values range from -0.01 ≤ r ≤ 1. These values were classified and interpreted as weak, moderate, strong according to values -0.01 < r ≤ 0.3, 0.3 < r ≤ 0.5, 0.5 > r, respectively.

Secondly, to clarify interdependencies between changes in test scores across time, a correlation matrix of the RCIs was created (see Figure 2). All correlation values (excl. autocorrelations) ranged from −0.01 to 0.95 (see Supplementary Table 1). Each subtest correlated the most with the same subtest at the other follow-up timepoint (ie, 6 months and at 1-year follow-up), with strong effect sizes (ie, correlations > 0.5). In addition, changes on the COWA test were weakly to moderately correlated with changes in the trail making and HVLT-R subtests, respectively. By contrast, changes in verbal memory (ie, HVLT-R) were moderately correlated with the trail making subtests. Finally, strong correlations were observed between changes in the trail making scores, even across timepoints of follow-up. In other words, changes in the trail making test seemed to be most robust across the different follow-up timepoints.

**Figure 3.**
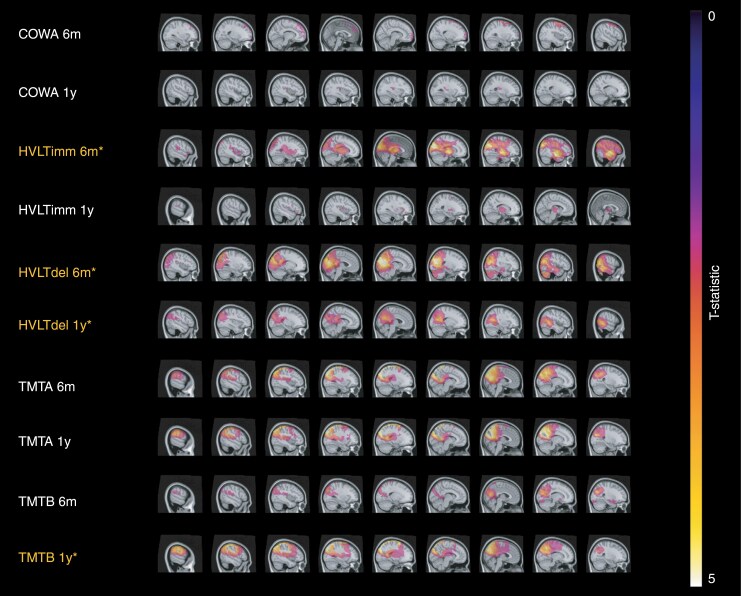
T-maps with significant clusters of higher doses associated with more cognitive decline *Note*. Voxel-wise T-statistics are presented for all voxels that reached significance at p_uncorr_ < 0.05 (at both peak- and cluster-level), with a color range set at 0 ≤ T ≤ 5. The higher the T-value, the stronger the association between RT dose and RCI scores. Test names that are indicated in white (in column on the left) reached significance at both voxel- and cluster level of p_uncorr_ < 0.05. Test names indicated in yellow additionally reached FWE-corrected significance at cluster level p_FWE_ < 0.05.

Based on the voxel-wise analyses, extensive clusters (ie, significant at cluster level) were encountered for most of the tests (see Figure 3). As a result, for each test, at least one cluster reached significance at uncorrected cluster p-level. However, after FWE-correction at cluster level, only the HVLT-R immediate at 6 months, HVLT-R delayed at both timepoints, and TMT B at 1 year, reached significance, showing the largest clusters. One should note that the cluster sizes of the TMT A were also large and were only borderline non-significant at FWE-corrected cluster level. By contrast, the highest significance at peak (ie. voxel) level was encountered for the TMT A at each timepoint, and the TMT B at 1-year follow-up (with T ≥ 6.81, *P*≤ .0006), followed by the HVLT-R immediate and delayed at 6 months (with *T* = 5.30, *P* = .0002). In other words, the decline in both HVLT-R and TMT subtests were strongly associated with voxel-wise dose (either at cluster or peak level), albeit the extent of the significant clusters, as well as the focal peak significance differed between the subtests and timepoints.

Regarding the decline in immediate verbal memory (HVLT-R immediate) at 6 months follow-up, significant dose effects were mainly detected in the temporal superior and middle gyri, occipital superior, and middle gyrus, with the highest significance at voxel level in the cuneus and small part of the optic radiations. The largest parts were located in the right hemisphere, but also extending to equivalent left cortices. Dose effects to similar regions were significant for decline in delayed verbal memory (HVLT-R delayed recall) at both 6 months and 1-year follow-up. By contrast, clusters found for immediate recall at 1-year follow-up, albeit not significant at FWE-cluster level, covered a few smaller and different locations (the left frontal superior gyrus, insula, putamen, thalamus, pallidum).

For changes in processing speed (TMT A), a large cluster (albeit borderline non-significant at FWE-cluster level) was found involving the bilateral pre-, para- and post-central regions, right precuneus, cuneus, and calcarine fissure. The highest voxel significance was encountered in the left postcentral gyrus and parietal superior gyrus. This effect was found at both 6 months and 1-year follow-up, and was similar for cognitive flexibility (TMT B) at 1-year follow-up, for which the highest voxel significance was extended even further to the left pre- and para-central gyri and left parietal inferior cortex, for which the entire cluster reached significance at FWE-cluster level. Clusters related to cognitive flexibility at 6 months follow-up, partly overlapped with or were adjacent to these previously mentioned regions (ie, bilateral (pre)cuneus, and occipital superior and middle gyri), but were non-significant at FWE-cluster level.

For verbal fluency, only small clusters were encountered based on the COWA test at both 6 months and 1-year follow-up, which were located in the left and right-sided frontal gyri (superior, middle, pre-, and postcentral gyri).

## Discussion

To our knowledge, this is the first study investigating the spatial impact of radiation dose on cognitive changes after cranial radiation therapy, using a voxel-based approach. This method allows us to assist in determining new eloquent (ie, functionally crucial) brain areas at risk that need to be spared from cranial radiation. After correcting for practice effects, cognitive performance on average remained relatively stable on most subtests. Higher proportions of reliable decline in performance were observed after 6 months (in 1/6 to 1/4 patients across tests), compared to 1-year follow-up. Yet, reliable decline in cognitive flexibility was still observed from baseline to 1-year follow-up in about 1 out of five patients. Based on the voxel-based imaging analyses, higher cranial radiation doses were most strongly associated with both short- and long-term changes in verbal memory, processing speed, and cognitive flexibility, whereas associations with changes in verbal fluency were non-significant. Brain regions involved in decline in memory were mostly located in the bilateral temporal and occipital lobes. Regarding decline in processing speed and flexibility, the affected regions were mainly located in areas controlling movement, somatosensory and visuospatial processing, depending on the timepoint and subtest. Overall, these results suggest both focal as well as more widespread dose effects on cognitive changes and neurotoxicity.

### Dynamics in Cognitive Decline After Cranial Radiation

The current study suggests relatively stable performance, with a minority, i.e. about 20-25% of patients, exhibiting “significant” changes in cognitive tests based on the Reliable Change Index (RCI). These findings support earlier evidence from Karunamuni et al. (2020) showing relatively stable profiles.^43^ Similarly, a meta-analysis by Lawrie et al. (2019) demonstrated very limited evidence for group differences between radiation dose- or type-defined subgroups.^44^ However, as this analysis included only nine studies, the authors strongly advocated for the standardization of cognitive assessments across trials, to reduce uncertainty about the absence of radiotoxic effects. While the interpretation of RCIs with a 95% confidence interval as a “reliable change” assisted in identifying individuals with the most pronounced cognitive alterations, it is important to acknowledge that also smaller changes are still clinically meaningful. We addressed this concern by utilizing a permutation-based linear regression model, allowing us to model cognitive change as a continuous variable, spanning the entire range of observed scores and radiation doses. With this approach, we show local dose associations with cognitive decline, both at 6 months as well as 1 year follow-up, suggesting spatial differences in toxic and functional dose effects.

In line with our previous meta-analysis,^45^ we encounter reliable decline in performance on the Trail Making Test B as the most prominent and stable decline across tests. One should notice that in post-hoc analyses, pre-treatment scores show rather limited levels of impairment in the trail-making tests, compared to the memory and fluency tests (see Suppementary Figure 1). This suggests that the baseline performance versus the pattern over time (stability (eg, fluency) versus decline (eg, memory, cognitive flexibility)) are function-specific, depending on lesion locations, dose distributions, and targeted areas. Based on the current study, decline in performance was mostly observed shortly after treatment (ie, at 6 months), while on average, patients improved by the later timepoint. This is in line with earlier findings.^46^ However, other studies indicated stable functioning in patients with LGGs after 6 years, while performance progressively deteriorated after 12 years.^24^ Besides the shorter duration of follow-up, the inclusion of patients with higher-grade tumors, of whom cognitive performance could decline much faster due to both the tumor progression, and changes in treatment in the past decade (eg, higher RT dose fractions in earlier treatment regimens, and the use of 2D RT/whole-brain RT^24^) can explain these differences. The current study focused on relatively short-term effects (ie, 1-year follow-up). To know how the cognitive trajectories further develop (after 1 year) and to address non-linear, longer follow-up of a larger sample of patients is required. For these reasons, the current study will be continued in recruitment and data acquisition at later timepoints. At this point, non-linear effects can only be hypothesized, for which in post-hoc visualization, significant decline in RCIs could start from a radiation dose to the test-specific brain area of 7.73Gy onwards (see Supplementary Figures 2 and 3). This suggests that even relatively small increases in radiation dose may be associated with meaningful cognitive decline, underlining the need for careful dose planning even at lower dose levels.

### Focal Versus Global (network-based) Radiation Effects—How to Look at Future OARs

Based on the voxel-wise statistics, reaching significance at peak level, we encountered significant voxels of which radiation doses were associated with cognitive decline. More specifically, we found significance for decline in verbal fluency in the frontal gyri; for decline in verbal memory in the right cuneus, optic radiations, and calcarine fissure (as well as left frontal superior gyrus, insula, and putamen in case of immediate recall at 6 months, and the right middle temporal gyrus of delayed recall at 1 year); for decline in processing speed in the left postcentral gyrus and parietal superior gyrus, which was similar for cognitive flexibility at 1-year follow-up, for which highest voxel significance was extended even further to the left pre- and para-central gyri and left parietal inferior cortex. Clusters related to cognitive flexibility at 6 months follow-up, partly overlapped with or were adjacent to the earlier regions related to immediate verbal memory (ie, bilateral (pre)cuneus, and occipital superior and middle gyri). Across the tests, voxel-wise associations (at peak level) were strongest with changes in processing speed at 1-year follow-up, followed by cognitive flexibility (at any time), and then verbal memory at 6 months follow-up. One could argue that the focal peak-level effects associated with these changes could be defined as crucial regions at risk. The hypothesis of sparing regions such as the left postcentral gyrus and parietal superior gyrus, for preserving processing speed and cognitive flexibility at 1-year follow-up, is supported by an earlier study demonstrating similar dose effects in the parieto-occipital region on slower visuomotor processing in meningioma patients.^47^ Additionally, the fact that cluster-based effects on both processing speed and cognitive flexibility also covered occipital regions, matches closely with these previous results. This suggests the encountered visuomotor areas and wider scale networks to be recruited for these specific subtests, which might have become less efficient after radiation treatment. Similarly, we might be able to preserve (verbal) memory, by avoiding the more focal structures in the radiation planning which showed significance at peak level. Even though focal structures such as the hippocampus and middle temporal gyrus have most consistently been associated with memory performance previously,^12^ also connectivity between these and other brain areas is increasingly shown to play a role in memory outcomes.^48–50^ Interestingly, significant clusters that were repeatedly encountered across the tests, including the precuneus and areas surrounding the parieto-occipital fissure,^51^ partly align with the posterior part of the default mode network. This network has widely been associated with internally directed attention as well as memory skills.^52^ Hence, the impact of radiation dose to specific structures on cognition not only depends on their function under healthy conditions but also on shifts in functional localization, which might be driven by functional or structural subnetworks. Also, the phenomenon of diaschisis could explain how initial damage to the encountered occipital regions could lead to disruption of neural pathways connecting with core memory systems. However, most radiation avoidance studies to date have mainly focused on avoiding high dose to the hippocampus, mainly in patients receiving whole-brain irradiation for metastases (eg, lung cancer patients).^53,54^ Although some of these studies have evidenced promising results, with limited decline in executive functioning and memory,^55^ without^56,57^ or with memantine treatment,^58,59^ it still remains unclear whether these effects are strongest by avoiding the hippocampus specifically, and whether they are generalizable to daily life. Previous dosimetric studies largely focused on delineated structures, while the current study applied a voxel-based approach, suggesting both focal as well as more global (network-related) dose effects. For decline in memory, processing speed, and flexibility (1), as well as fluency (2), one can assume the involvement of temporal-parietal (1), and frontoparietal networks (2), respectively. Such involved networks seem to overlap with the encountered significant larger-scale clusters per subtest. Based on our results at voxel-peak significance level specifically, one could suggest the area surrounding the parieto-occipital fissure to be a potentially important OAR for cognitive skills involved in the TMT and HVLT (see Supplementary Figure 4 for clusters thresholded at peak significance T > 4). This area is consistently right-sided, while change in the TMT scores also cover a part of the left-sided superior parietal lobule. Even though these focal (peak) effects exist, the fact that changes in cognitive flexibility at 6 months were associated with similar regions as decline in immediate verbal memory, as well as the fact that decline by 6 months versus 1-year follow-up yielded different brain areas, suggest that interactions exist between diverse brain areas. The hypothesis therefore arises that the focal peak significances of dose effects indicate specific eloquent brain regions that are possibly located at a core location in the brain network involved in a certain function. The question of whether the regions encountered in this study are indeed the most crucial hubs (or whether they became hubs throughout radiation therapy) involved in cognitive decline after radiation, needs further investigation. We suggest to combine voxel-based and network-based analyses in future analyses, to further refine definitions of future OARs for radiotherapy planning. This data-driven approach could more precisely estimate voxel-specific effects and assist in defining functional *regions at risk* and their boundaries, rather than how OARs are defined to date, ie, by anatomical boundaries.

### Neurobiological Mechanisms, Neuroimaging of Radiotoxicity, and Clinical Developments

Spatiotemporal vulnerability of specific brain areas to radiotoxic effects is still largely under debate.^3^ One of the existing hypotheses posed by Gibson and Monje (2019), is that microglia are activated by cancer treatment, which is followed by alterations in neural precursor cells as well as mature cells. These time-dependent mechanisms involve intercellular interactions (including oligodendrocytes, microglia, astrocytes, and neurons).^60^ The earlier hypothesis of the specific vulnerability of regions where neural stem cells arise, ie, mainly the hippocampus and subventricular zone, is slowly being replaced by the hypothesis of spatial distributions on diverse cell types. Depending on the tumor location, its progression, and the applied radiation, diverse neurotoxic biological mechanisms are possibly induced (eg, inflammation, demyelination, . . . ).^61–64^ In addition, recent network-based studies now suggest some initial evidence for hub-specific vulnerability to radiotoxicity.^30,65^ Future studies might consider the use of multimodal neuroimaging and incorporation of pre-treatment features of patients,^43^(eg, pre-existing white matter damage).^66^ Finally, some first findings on pre-treatment serum measurements suggest that lower levels of ApoJ, ApoE, or ApoA proteins and higher levels of amyloid beta (Aβ 1-42) could increase the risk of developing radiation-induced cognitive impairment.^67^

^717247^To better preserve healthy tissue and cognitive functioning in adult brain tumor patients, defining OARs may need to shift toward more network-based and voxel-based approaches, improving current prediction models for cognitive changes after cranial radiation (as earlier discussed). Once critical brain areas are identified, patients may gain further advantages from modern radiation techniques, such as VMAT^68^ and proton therapy,^69^ which allow for more precise dose distribution and enhanced sparing of healthy tissue. Whether data-driven voxel-based analyses converge with network-based hub effects on cognitive changes throughout treatment requires future advanced imaging studies. Furthermore, other adjuvant treatments such as chemotherapy^70^ and most recent treatment developments, involving immunotherapies^71–73^ and targeted therapies,^74,75^ also yield the new scientific question on potential interactions with radiotoxicity and changes in cognitive functioning throughout treatment.

Although our current approach is not yet directly implementable, it provides a critical proof-of-concept of the feasibility and value of voxel-based risk estimation. By taking a function-driven perspective, and applying a voxel-based analysis, we reveal that the regions most vulnerable to radiation-induced cognitive decline do not align with typical anatomical OARs. Even more, in post-hoc calculations, we demonstrate that of the current OARs,^35^ only four overlap for at least 50% with voxels significantly associated with HVLT recall/verbal memory (ie, amygdala, fornix, thalamus, caudate nucleus) (see Supplementary Figure 5 and Table 2). By contrast, less than half of the hippocampus was overlapping with these significant voxels (and even fewer voxels when looking at the other tests). One should note that RT treatment plans in this cohort were subjected to RT dose constraints, ie, a RT max. dose of 7.3 Gy in EQD2 to 40% of the bilateral hippocampi.^76^ Across TMT and HVLT tests, the posterior part of the corpus callosum repetitively overlapped with the significant voxels (ie, ±36–49%). The current data-driven approach underscores the need to move toward functionally informed treatment planning and provides empirical evidence that cognitive risk is more accurately captured at the voxel level. Our work offers a new methodological perspective on software development integrating voxel-wise functional risk maps into clinical radiation planning. Such implementation of high-resolution risk maps in planning might additionally benefit from integrating both voxel-level dose mapping as well as individualized functional brain imaging, holding potential to individually tailor planning to avoid cognitive decline. However, before clinical implementation, further large-scale studies are needed to validate our results.

**Table 2.** Statistics and Regional Information Related to the Significant Clusters per Subtest

Cognitive test	Follow-up	T(p_uncorr_) peak-level	n of sign. clusters at p_uncorr_ < 0.05 (min. k)	n of sign. clusters at p_FWE_ < 0.05 (min. k)	Regions involved
COWA	6m	2.15 ≤ T_uncorr_≤ 3.80 (0.0004 ≤ p_uncorr_≤ 0.0186)	4 (k ≥ 318)	0	Largest cluster right-sided: frontal superior and middle gyrus, (pre- and) postcentral gyrus; left frontal superior and middle, precentral, gyrus
	1y	1.93 ≤ T_uncorr_≤ 2.21 (0.0162 ≤ p_uncorr_≤ 0.0236)	2 (k ≥ 1097)	0	One cluster left-sided (SLF/CST crossing region, insula, Heshl gyrus); one right-sided in the frontal superior gyrus.
HVLT-R immediate	6m	T_FWE_ = 5.30 (p_FWE_ = 0.0002); 2.36 ≤ T_uncorr_≤ 2.88 (0.0044 ≤ p_uncorr_≤ 0.0110)	3 (k ≥ 2904)	1 (k = 693217)	Largest cluster right-sided, extending to the left cortices: temporal superior and middle gyrus, occipital superior and middle gyrus, cuneus small part of the optic radiations (cluster right superior frontal gyrus, orbital part at p_uncorr_ < 0.05)
	1y	2.34 ≤ T_uncorr_≤ 3.11 (0.0008 ≤ p_uncorr_≤ 0.0072)	5 (k ≥ 978)	0	Highest voxel significance found in left frontal superior gyrus, orbital part, insula, putamen. Clusters additionally include left thalamus, CST, pallidum.
HVLT-R delayed	6m	T_FWE_ = 5.05 (p_FWE_ = 0.0002); 2.34 ≤ T_uncorr_< ≤0.56 (0.0014 ≤ p_uncorr_≤00.0168)	3 (k ≥ 1613)	1 (k = 599674)	Largest cluster right-sided, extending to the left. Again right cuneus and calcarine fissure show highest voxel significance. (2 clusters left cerebellar, paracentral lobule at p_uncorr_ < 0.05).
	1y	T_FWE_ = 3.92 (p_FWE_ = 0.0004); 2.16 ≤ T_uncorr_≤ 2.43 (0.0086 ≤ p_uncorr_≤ 0.0154)	4 (k ≥ 1566)	1 (k = 335071)	Largest cluster right-sided, extending to the left. Again right cuneus and calcarine fissure show highest voxel significance. Also the right middle temporal gyrus now shows high voxel significance.
TMT A	6m	T_uncorr_ = 6.78 (p_uncorr_ = 0.0052);	1 (k = 346346)	0	One large cluster (borderline non-significant), involving the right precuneus, cuneus and calcarine fissure, left and right pre- and paracentral regions. Highest voxel significance in left postcentral gyrus and superior parietal gyrus.
	1y	T_uncorr_ = 7.11 (p_uncorr_ = 0.0004)	1 (k = 366225)	0	One large cluster, borderline non-significant.
TMT B	6m	2.81 ≤ T_uncorr_≤ 3.87 (0.0032 ≤ p_uncorr_≤ 0.0094)	2 (k ≥ 23461)	0	Bilateral clusters in (pre)cuneus, occipital superior and middle gyri. Highest voxels significance in right cuneus and occipital superior gyrus.
	1y	T_FWE_ = 6.81 (p_FWE_ = 0.0006);	1 (k = 412592)	1 (k = 412592)	Largest cluster left-sided, extending to the right, involving the (pre)cuneus, occipital superior gyrus. Highest voxel significance in left pre-para-postcentral gyri, left parietal inferior and superior cortex.

*Note.* k = cluster size indicated as number of voxels. T_FWE_ = T-value (and its uncorrected p-value) at peak level within cluster that was significant at p_FWE_ < 0.05. T_uncorr_ = T-value (and its uncorrected p-value) at peak level within cluster that was significant at p_uncorr_ < 0.05.

### Limitations and Strengths

Even though this study innovatively explored the longitudinal voxel-wise impact of cranial radiation on cognitive functioning, some limitations must be considered. First, certain methodological choices may have influenced the results. We selected the physical 3D dose distribution images for analyses, as they provide a direct, untransformed representation of radiation exposure. However, the biological response of diverse brain tissues is not homogeneous. An alternative approach could involve using equivalent doses in 2Gy fractions (EQD2), also accounting for differences in fractionation schemes. Yet, this method requires assumptions about tissue repair, and as the α/β ratio varies across the brain tissue types, applying a single ratio across the entire brain may oversimplify the biological response. To evaluate this, we compared the inter-subject variability between EQ2D and physical dose maps, showing consistent patterns. This suggests converting the physical dose to EQD2 is of second order compared to the absolute variation of the dose levels. For preprocessing we applied 2 mm Gaussian smoothing. As radiation doses are considered as being robust and precise at the voxel level, this minimal level of smoothing was chosen, to minimize potential smoothing effects on the results. However, voxel-wise studies often employ larger smoothing kernels, to reduce voxel-specific uncertainty. We validated this by comparing results at 2 mm and 5 mm, which confirmed similar distributions of significance.

Second, the GTV volume was extracted from the radiation plans in the analyses, to focus on the voxel-wise impact of radiation on tissue that was considered to be “healthy.” Even after excluding these voxels, at least 83 data points were analyzed for each voxel. The standard deviation map showed uniform variability across the brain (see Supplementary Figure 6). Although the sample size remained adequate with consistent variability, we cannot rule out the additional impact of radiation to the tumor on cognitive changes throughout time, nor of the tumor volume/type/location itself. In addition, other confounders such as the use of chemotherapy (temozolomide [TMZ] vs procarbazine hydrochloride, lomustine [PCV]), anti-epileptic drugs, edema, IDH-mutation, etc. can have played a role in RCI scores. Unfortunately, these features are intrinsically interdependent in neuro-oncological populations, which complicates comprehensive modeling of these factors into one predictive model.

Before selecting the final model for predicting voxel-wise radiation doses, we tested associations between RCI scores, radiation subtype, tumor subtype, tumor volume, and peak doses using Mann–Whitney *U*-tests and Pearson correlations. Most of these tests were non-significant (*P* > .05), except for radiation subtype and tumor subtype (delayed recall at 6 months), tumor volume (immediate recall at 6 months), and delayed recall at 1-year follow-up (see Supplementary Table 3). Hence, we tested in post-hoc multiple regression analyses whether these confounders, ie, radiation type (photon vs. proton), tumor type (glioma vs. non-glioma), tumor volume, significantly predicted verbal memory RCI scores in addition to the radiation dose to the significant clusters from the voxelwise analyses. These analyses showed that verbal memory at longer follow-up (1 year) could additionally be affected by the tumor subtype (glioma vs. non-glioma) and delayed recall also by tumor volume (see Supplementary Table 4). In a validation analysis, we confirmed the negative dose associations between RCIs and RT doses to the significant clusters in the glioma subgroup only (see Supplementary Figure 7). Still, we cannot exclude the indirect effects of such confounders on memory changes. Additionally, factors such as education, age,^77^ household income, and work activities,^78^ can also play a role in individual vulnerability to decline in cognitive functioning.^79^ Finally, linear associations of RCIs—indicating the relative level of cognitive decline—with radiation doses were investigated. This approach was selected to avoid arbitrary group classifications (when subgroups would be created) and maximize statistical power. However, we recognize that our linear model may not fully capture the complexity of existing associations and that non-linear or outlier effects could still have influenced the results. To explore whether group comparisons would have yielded different results, we conducted a post-hoc group comparison (negative vs. zero-to-positive RCIs) using a permutation-based ANOVA, which yielded clusters similar to the linear effects (see Supplementary Figure 8). Still, there remains the possibility that (1) nonlinear dose-response patterns exist, and that (2) specific dose thresholds or cognitive performance cutoffs may be clinically relevant. To identify points of inflection in dose-response with (sub)clinical cognitive changes relationships, approaches such as spline regression or generalized additive models might be useful in studies with larger sample sizes and longer follow-up. Although practice effects were accounted for in the RCI calculations, relatively short testing intervals could still have positively affected the final RCI scores, particularly for memory.^45^ To mitigate this, alternate forms were implemented for both the COWA and HVLT-R at each timepoint, to minimize potential practice effects.

## Conclusion

This study demonstrated voxel-wise associations between decline in cognitive performance and cranial radiation dose. Decline was mostly observed at 6 months follow-up, mainly in processing speed, cognitive flexibility, and immediate verbal recall. After 1 year, still about 1 out of 5 patients demonstrated reliable decline in cognitive flexibility. The voxel-wise analyses demonstrated higher doses to frontal gyri, occipital and temporal regions, occipital and para-central areas, to be associated with short- and long-term decline in verbal fluency, verbal memory, and processing speed and flexibility, respectively, with cluster effects for the latter three outcomes. Our findings suggest specific local as well as more global network-based vulnerability to cranial radiation. Bearing temporal-occipital and fronto-parietal networks in mind as possible radiation-affected networks, the question arises whether the encountered regions based on the voxel-wise analyses are indeed specifically vulnerable due to their crucial position in brain subnetworks. These initial voxel-wise findings could assist in re-defining key OARs (eg, right cuneus, calcarine fissure, left postcentral, and superior parietal gyrus) and assist in building toward a network-based perspective, preserving cognition in future RT treatment planning.

## Supplementary material

Supplementary material is available online at *Neuro-Oncology* (https://academic.oup.com/neuro-oncology).

noaf114_Supplementary_Material

## Data Availability

The data are not publicly available due to the local GDPR regulations and approval of the local ethical committee to use the data only locally. After a data-transfer agreement and additional approval of the local ethical committee, pseudonymized data could only be shared upon request.
