# Supplementary material for: Radiotherapy-induced neurocognitive decline among adult intracranial tumor patients: A voxel-based approach
Source: Neuro Oncol. 2025 May 4;27(10):2634–46. doi: 10.1093/neuonc/noaf114 (PMC12833545; doi:10.1093/neuonc/noaf114)
Supplement: noaf114_Supplementary_Material [file noaf114_supplementary_material.docx]

**Supplementary materials**

**Preprocessing of scans and radiation dose distribution images**

Before initiation of the radiotherapy, delineation of gross tumor volumes (GTVs), encompassing the entire tumor volume along with the resection cavity, was performed by a radiation-oncologist specialized in neuro-oncology and reviewed by a second expert. This delineation relied on the anatomical MRI scan, which was co-registered with the CT scan for precise radiotherapy treatment planning ^1^. The 3D radiation dose maps from Eclipse, Varian Palo Alto (photon beam) or Raystation, Raysearch, Sweden (proton beam) were converted from DICOM to NIfTI-format using the MATLAB-based software OpenREGGUI^2^. These were re-oriented to match the original position of the converted CT- and MRI-scans and the radiological convention. Physical 3D Dose distribution images were rigidly co-registered and resampled onto the CT-scan grid. When the radiation treatment included multiple radiation plans, the delivered 3D RT dose was calculated as the weighted sum of the individual 3D dose distributions, corresponding with the effectively delivered physical doses.

To enable spatial standardization of all radiation plans and GTVs, the CT scans first underwent a reverse linear registration (rigid transformation) to align with the post-contrast T1-weighted MRI scan, of which the transformation matrix was consequently applied to the radiation dose and GTVs using B-spline and nearest neighbor interpolation, respectively. Once the radiation dose and GTVs were mapped on the T1-weighted MRI, spatial normalization of the images to a standard space could be performed. Specifically, following skull stripping of the images, the pre-contrast T1-weighted MRI underwent a non-linear registration involving rigid, affine, and deformable transformation to the population-based T1-weighted MRI brain template defined by the Montreal Neurological Institute and International Consortium for Brain Mapping (MNI-ICBM152). The same transformation was again applied to the radiation plans and GTVs with the abovementioned interpolations. All co-registration procedures were executed utilizing Advanced Normalization Tools ^3^ .

For subsequent voxel-wise analyses, the RT maps in MNI space were smoothed (2mm Gaussian Kernel). Finally, the GTV voxels were excluded (i.e. set to 0) from the RT dose image and voxel-wise analyses, in order to only retain the voxel-wise RT dose impact on healthy tissue and cognitive decline, while excluding tumor location-specific effects. These final images were used for voxel-based predictions of the reliable cognitive change scores.

**Definitions of significance at peak versus cluster-level**

Voxel-based statistics were reported with their significance at peak and at cluster level. The former tests whether the signal intensity (RT dose value) in a specific voxel is significantly associated with RCI scores, corrected for multiple comparisons across all voxels. Peak-level significance therefore highlights the strongest effects in very localized regions. Significance at cluster level by contrast, considers groups of contiguous voxels (clusters), and tests whether the size of a cluster is unlikely to occur by chance. It helps identify larger, spatially coherent patterns of dose effects, that may not show significance at individual voxel levels, but are significant as a group. The statistical significance threshold was set at a p-value of <0.05 at cluster-level.

Table 1. Correlation matrix with correlation values between RCIs across cognitive subtests

|  | COWA 6m | TMT A 6m | TMT B 6m | HVLT-R immediate 6m | HVLT-R delayed 6m | COWA 1y | TMT A 1y | TMT B 1y | HVLT-R immediate 1y |
| --- | --- | --- | --- | --- | --- | --- | --- | --- | --- |
| COWA 6m | 1 |  |  |  |  |  |  |  |  |
| TMT A 6m | 0.1417 | 1 |  |  |  |  |  |  |  |
| TMT B 6m | 0.1976 | 0.6791 | 1 |  |  |  |  |  |  |
| HVLT-R immediate 6m | 0.4221 | 0.2556 | 0.3623 | 1 |  |  |  |  |  |
| HVLT-R delayed 6m | 0.2401 | 0.3432 | 0.3156 | 0.4868 | 1 |  |  |  |  |
| COWA 1y | 0.5805 | 0.1789 | 0.1495 | 0.1080 | -0.0139 | 1 |  |  |  |
| TMT A 1y | 0.1170 | 0.9541 | 0.8524 | 0.2214 | 0.3548 | 0.1553 | 1 |  |  |
| TMT B 1y | 0.1337 | 0.9498 | 0.8771 | 0.2380 | 0.3260 | 0.1548 | 0.9132 | 1 |  |
| HVLT-R immediate 1y | 0.2641 | 0.2024 | 0.1446 | 0.6260 | 0.1472 | 0.2188 | 0.1619 | 0.1948 | 1 |
| HVLT-R delayed 1y | 0.1537 | 0.2434 | 0.1978 | 0.1133 | 0.7683 | 0.0348 | 0.2887 | 0.2813 | 0.1849 |

Figure 1. Waterfall plots of baseline z-scores


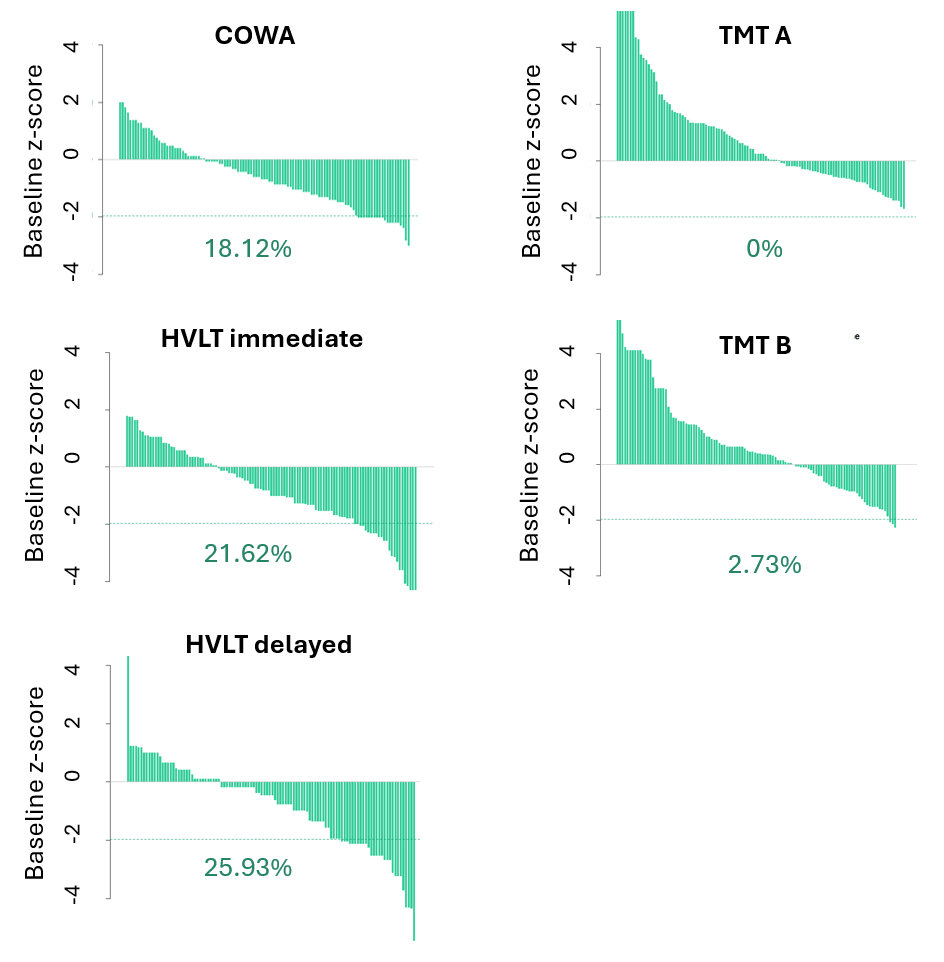


*Note.* Raw baseline scores were converted to z-scores based on test-specific normative data for the Controlled Word Association Test (COWA) ^4^, Trail Making Test (TMT) ^5^, and Hopkins Verbal Learning Test-Revised (HVLT-R) ^6^. A cut-off line to indicate test-specific impairment was presented at Z=-1.96.

Figure 2. Boxplots of radiation doses in patients showing decline (RCI<-1.96) versus no decline


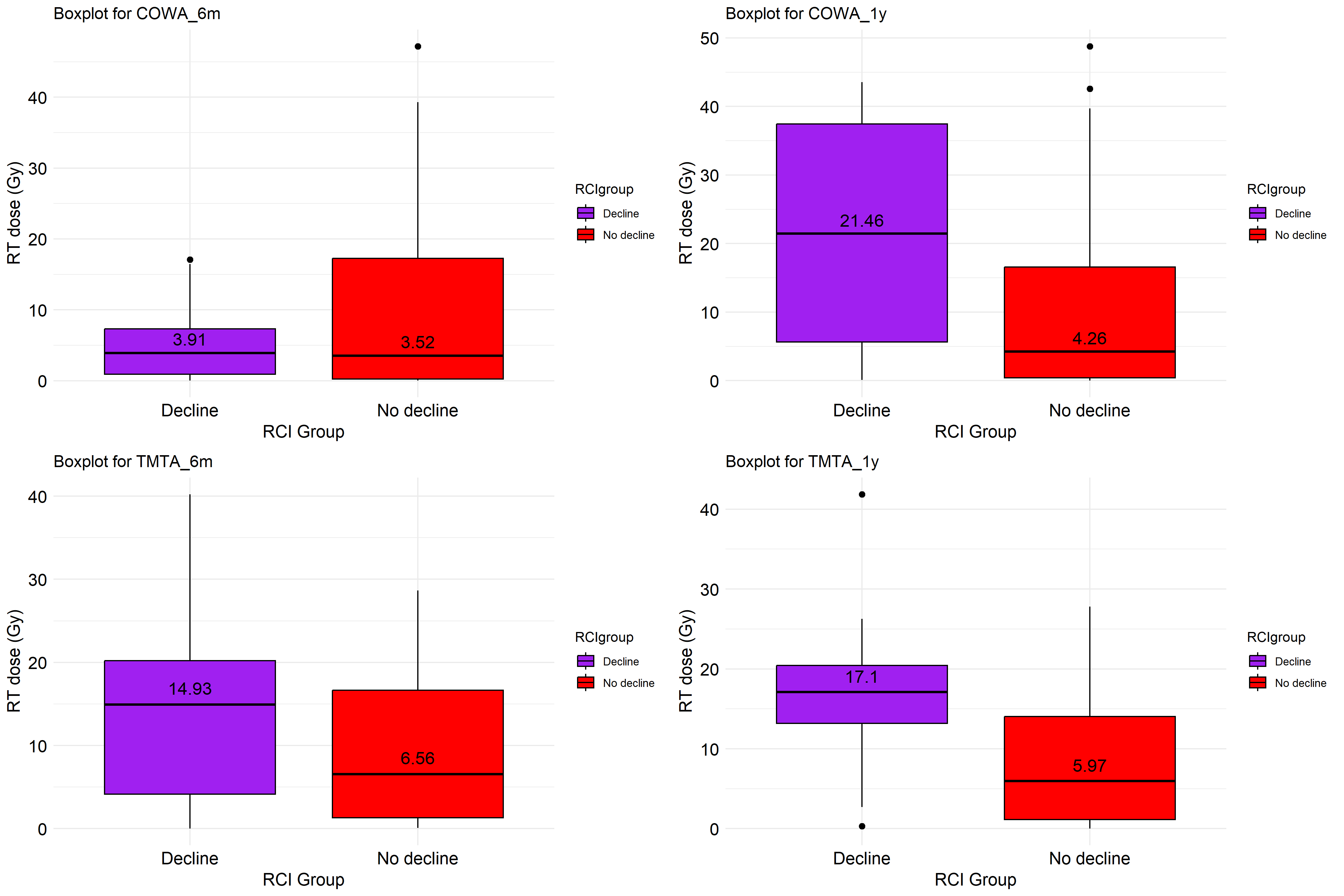


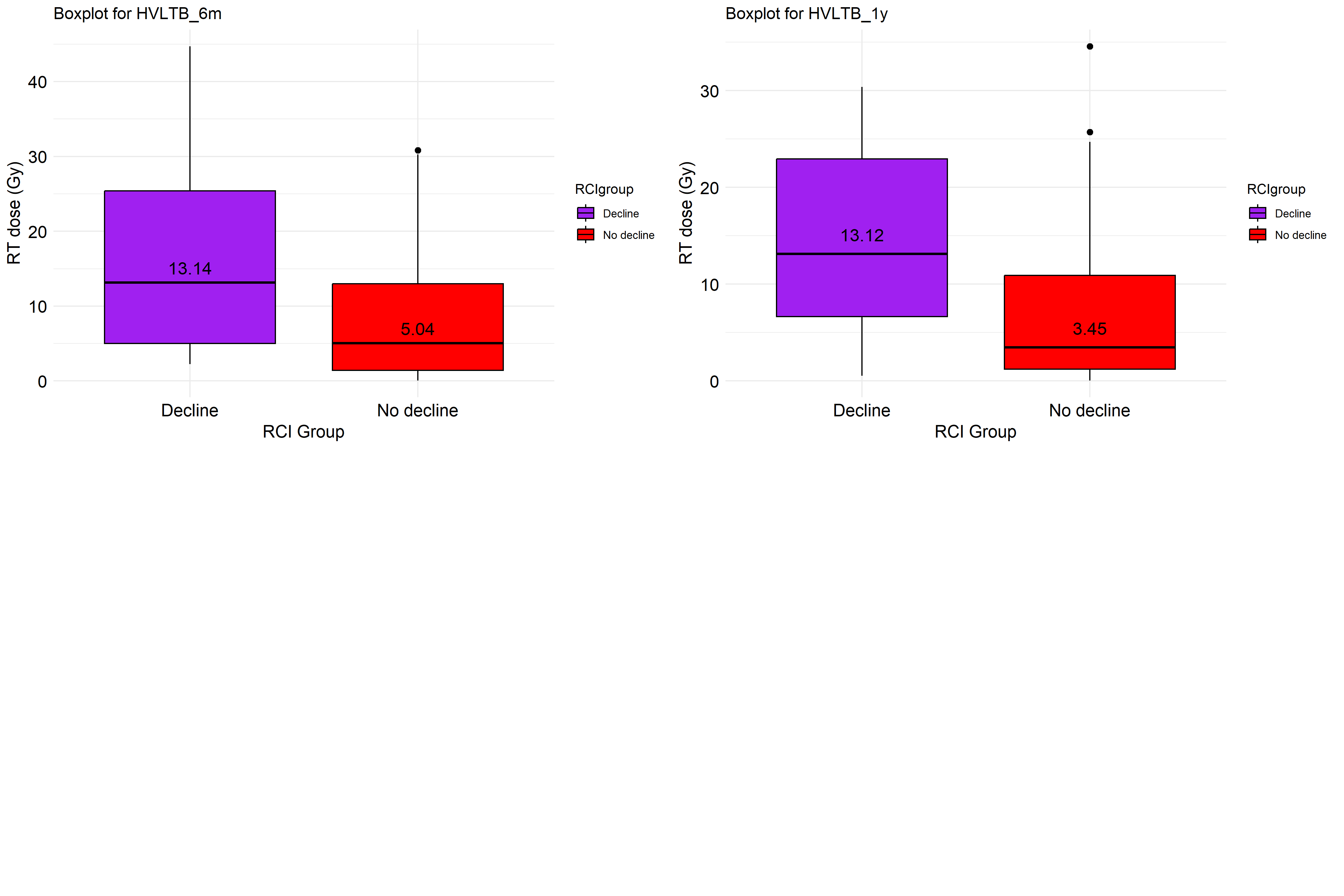

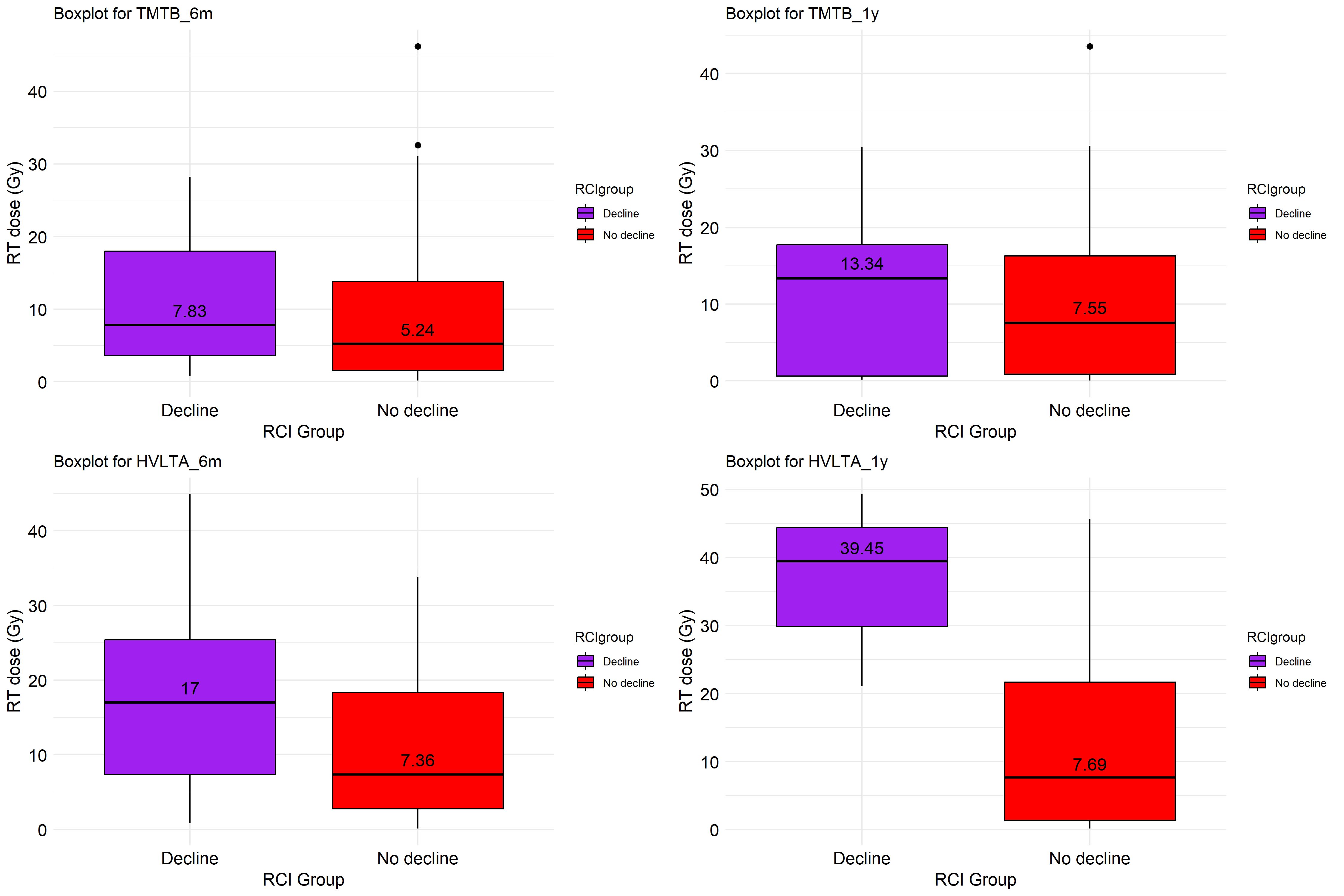


Figure 3. Scatterplots of RCI scores against RT doses in test-specific significant cluster


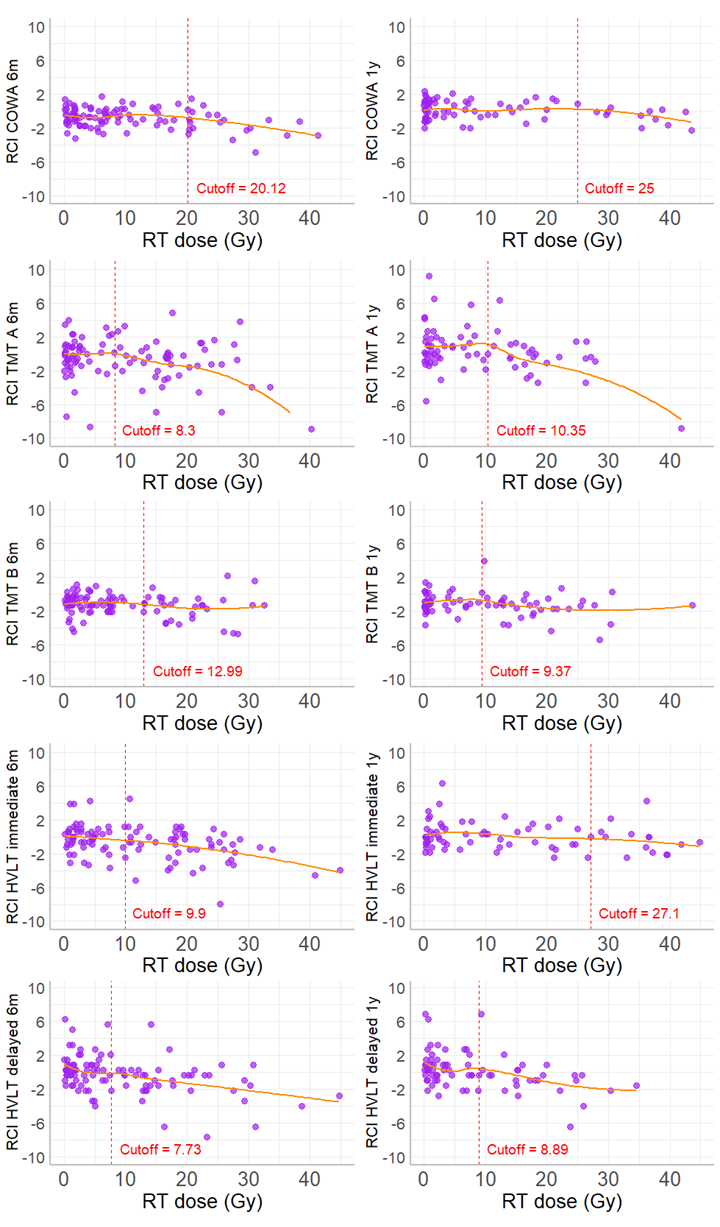


Note. A LOESS regression was performed to fit the datapoints nonlinearly. A cutoff line is drawn at the level of the last or second-to-last peak as the key inflection point, in tests with steep (i.e. HVLT, TMTA) and less steep declines (i.e. COWA, TMTB), respectively.

Figure 4. Thresholded map with significant voxels at level T>4 (peak-level)


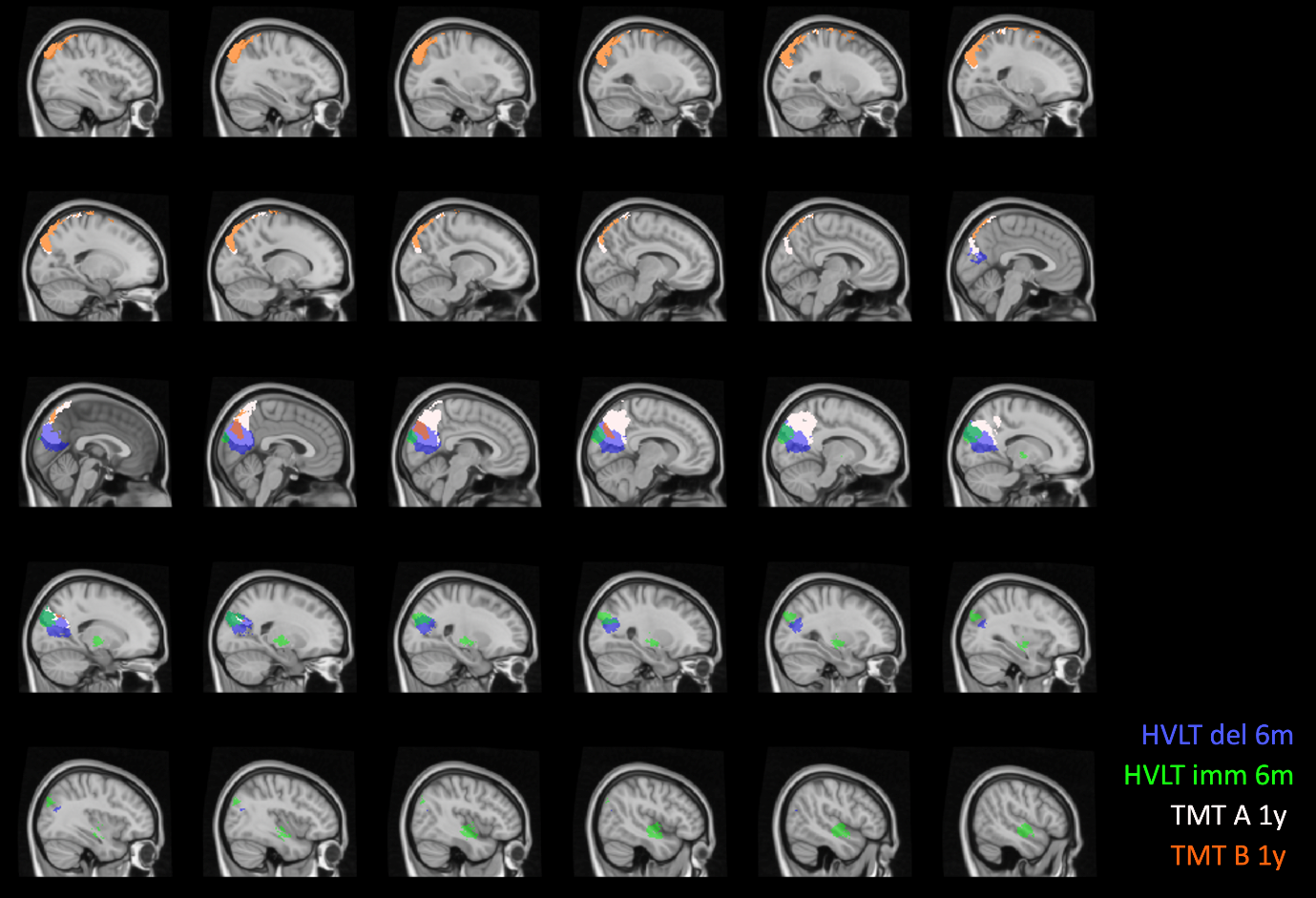


**HVLT del 6m
HVLT imm 6m
TMT A 6m/1y
TMT B 1y**


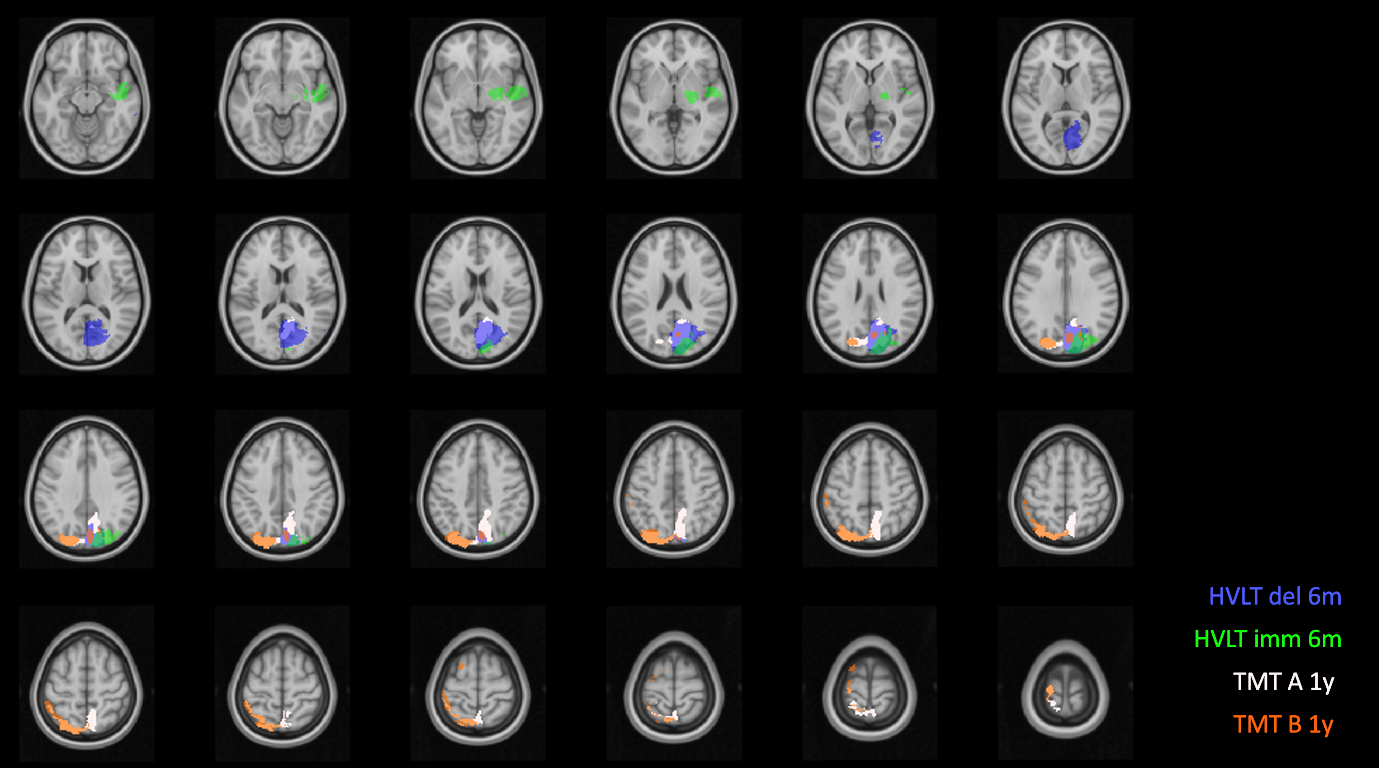


Note. At peak significance level of T>4, voxels related to decline in TMT A at 6months were almost fully covered by the voxels related to TMT A at 1year; therefore not added to the picture as a separate mask. For other cognitive tests, no voxels reached significance at T>4 peak level.

Figure 5. Voxelwise significance maps combined with standard neuro-oncological OARs


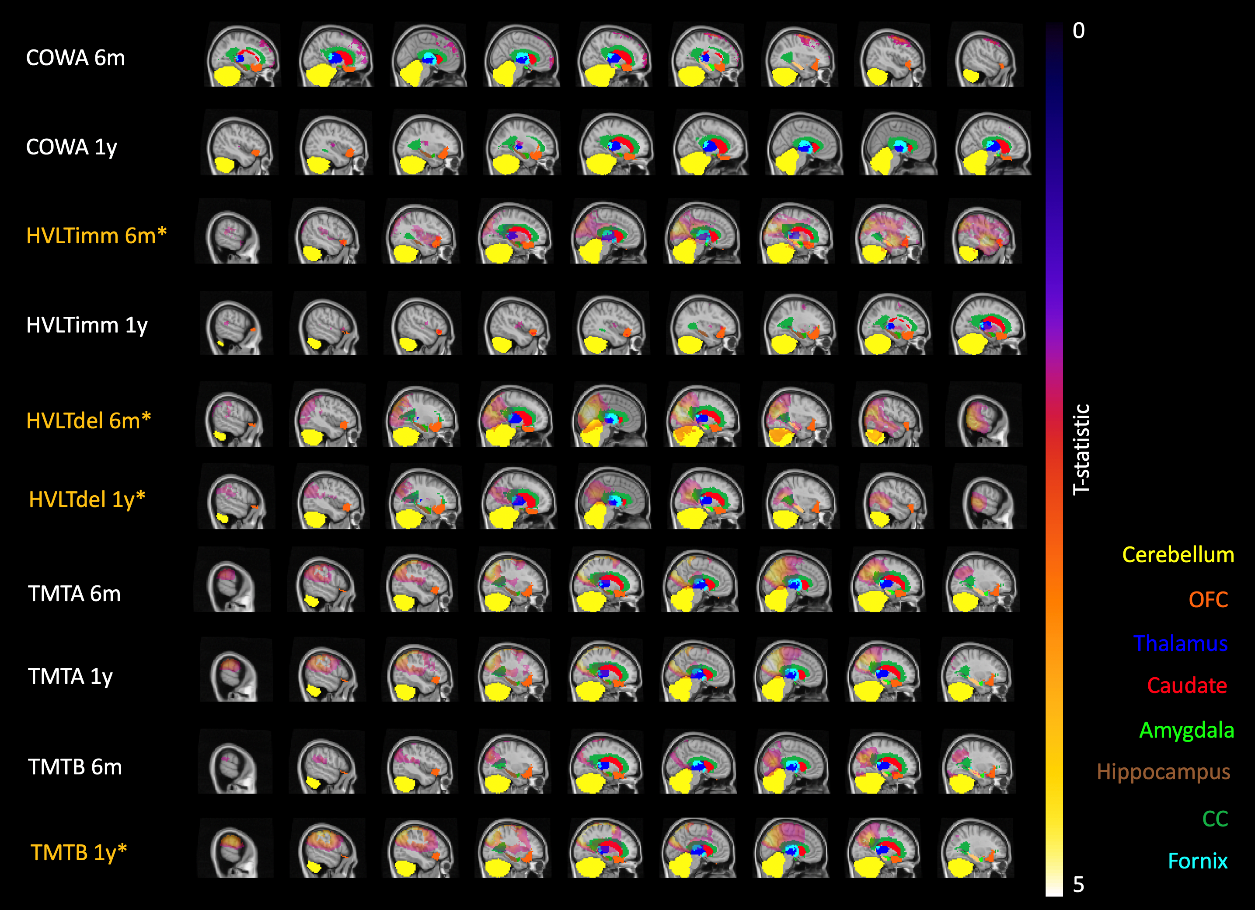


Table 2. Percentages of standard neuro-oncological OARs and significant voxels from the voxel-wise analyses (p<.05)

|  | OFC | amygdala | caudate | cerebellum | CC | fornix | thalamus | hippocampus |
| --- | --- | --- | --- | --- | --- | --- | --- | --- |
| COWA1y | 0,00 | 0,00 | 0,00 | 0,00 | 0,00 | 0,00 | 0,01 | 0,00 |
| COWA6m | 0,00 | 0,00 | 0,00 | 0,00 | 0,00 | 0,00 | 0,00 | 0,00 |
| HVLTimm1y | 0,03 | 0,00 | 0,00 | 0,00 | 0,00 | 0,09 | 0,31 | 0,00 |
| HVLTimm6m | 0,28 | 0,61 | 0,50 | 0,02 | 0,49 | 0,96 | 1,00 | 0,44 |
| HVLTdel1y | 0,00 | 0,00 | 0,03 | 0,01 | 0,39 | 0,39 | 0,30 | 0,11 |
| HVLTdel6m | 0,00 | 0,09 | 0,01 | 0,24 | 0,36 | 0,29 | 0,20 | 0,22 |
| TMTA1y | 0,00 | 0,00 | 0,10 | 0,01 | 0,37 | 0,38 | 0,29 | 0,04 |
| TMTA6m | 0,00 | 0,00 | 0,07 | 0,01 | 0,36 | 0,33 | 0,18 | 0,03 |
| TMTB1y | 0,00 | 0,00 | 0,30 | 0,00 | 0,44 | 0,37 | 0,27 | 0,03 |
| TMTB6m | 0,08 | 0,17 | 0,01 | 0,02 | 0,13 | 0,02 | 0,00 | 0,11 |

Note. Color scale: green versus red indicates low versus high percentage of overlap, resp.

Figure 6. Standard deviation map based on the dose distributions per voxel across participants


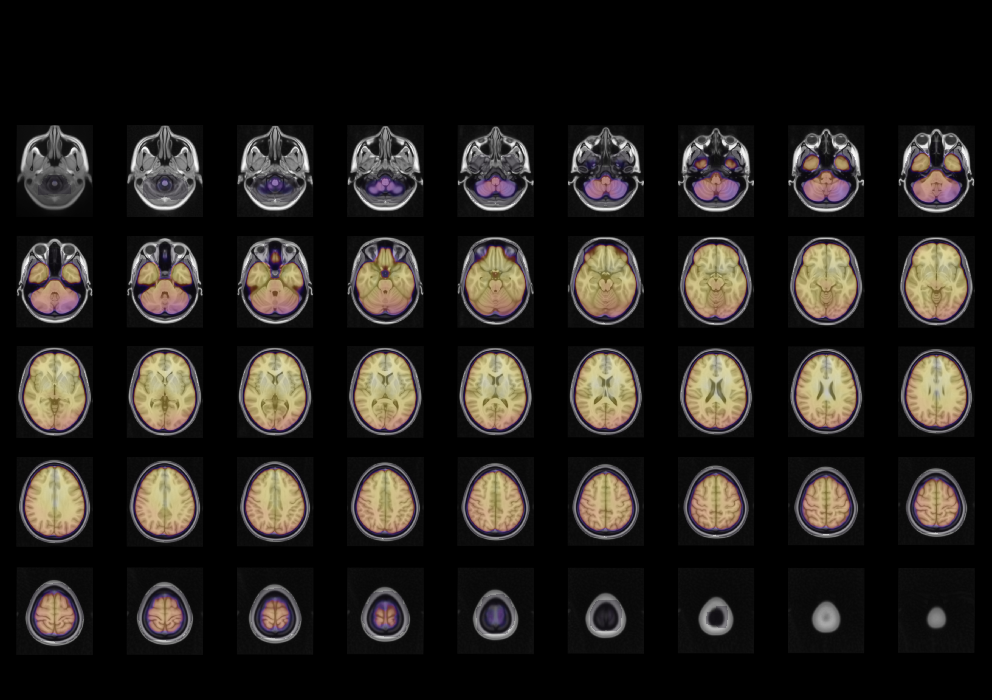


Figure 7. Scatterplots of RCI scores against RT doses in test-specific significant cluster – glioma patients only


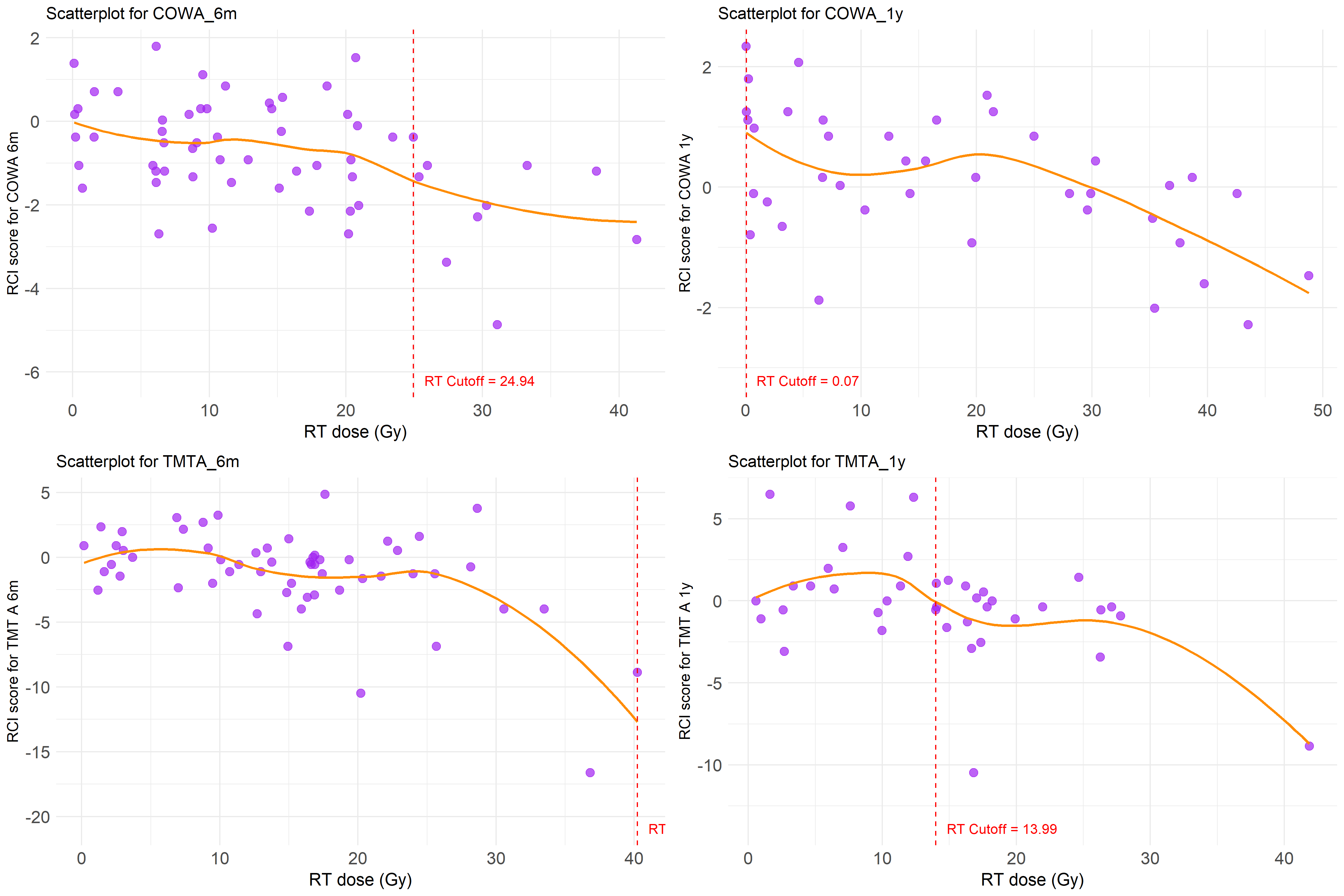

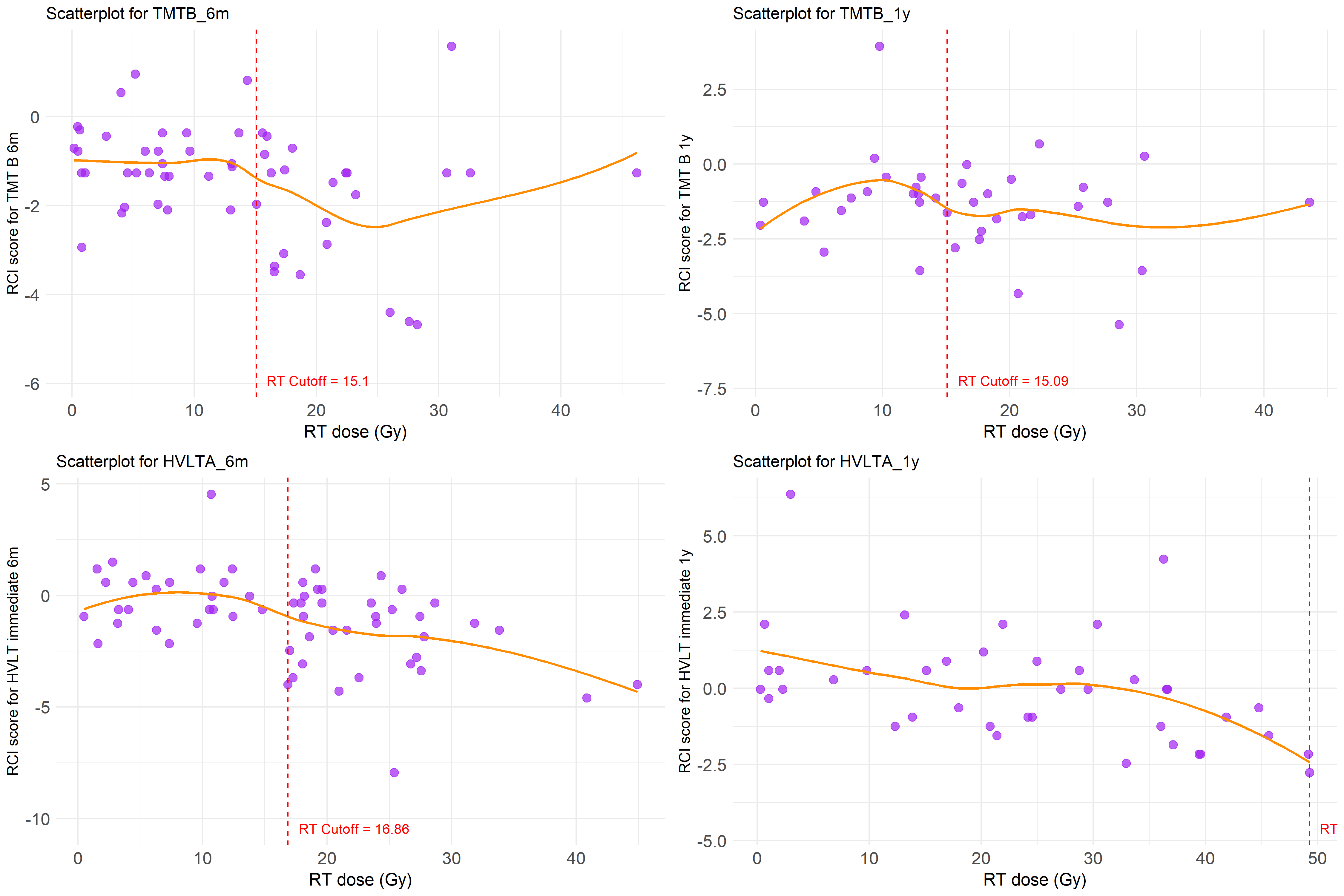


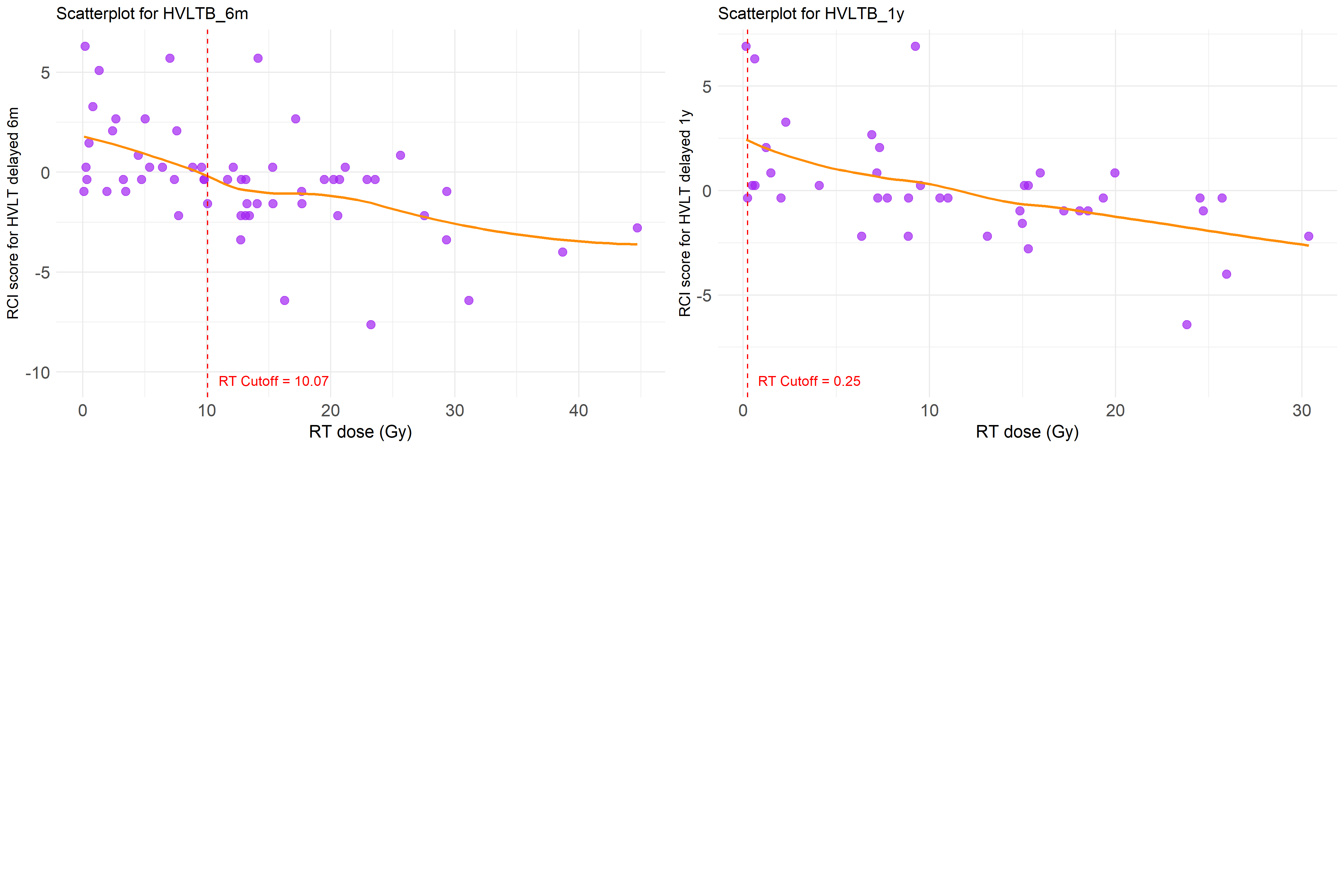


Figure 8. Comparison of linear versus categorical permutation-based statistics for RCIs of the Controlled Word Association Test (baseline to 6 months follow-up)


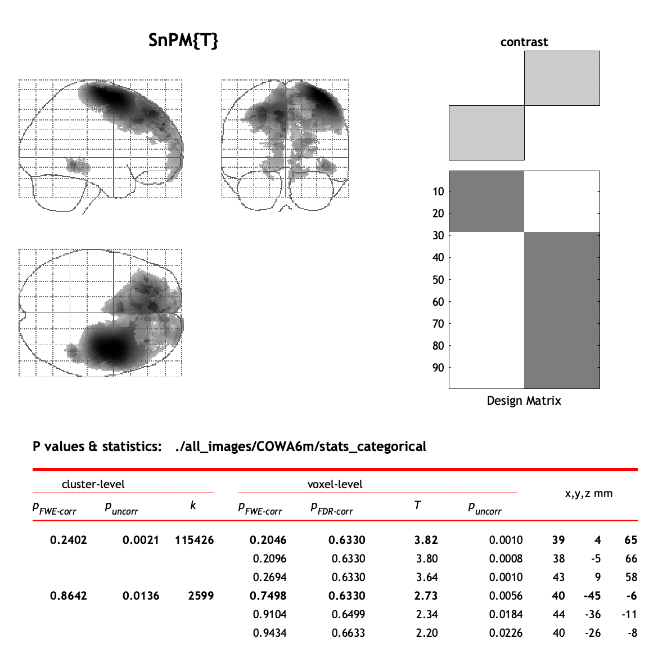

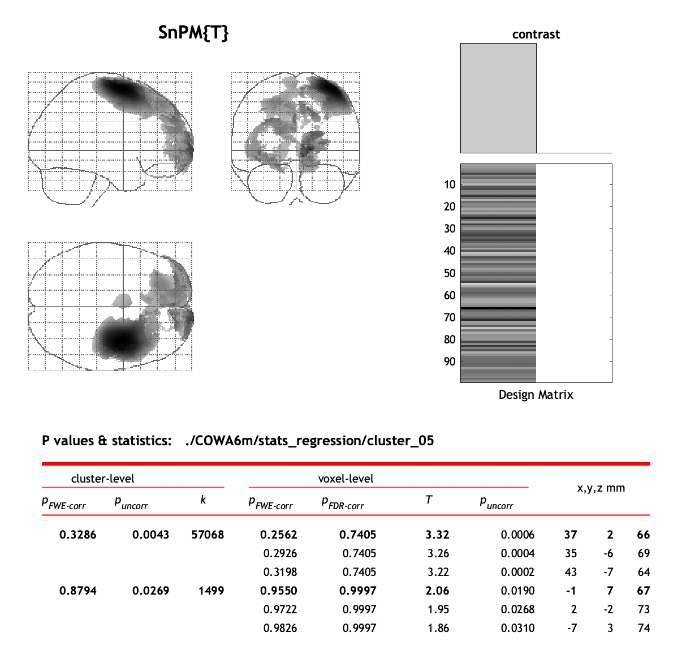


*Note.* Panel A depicts the significant voxels, based on the linear model that was applied in the manuscript. Panel B depicts significant voxels based on a categorical comparison, comparing patients reaching RCI$\leq$0 vs. RCI>0. As can be observed, spatial distributions of significance are comparable.

Table 3. Mann-Whitney U-tests and Pearson correlations of potential confounding factors and RCIs

Mann-Whitney U-tests (RCIs predicted by radiation subtype)

|  | COWA 6m | HVLT-R imm 6m | HVLT-R del 6m | TMT A 6m | TMT B 6m | COWA 1y | HVLT-R imm 1y | HVLT-R del 1y | TMT A 1y | TMT B 1y |
| --- | --- | --- | --- | --- | --- | --- | --- | --- | --- | --- |
| U-value | 1389 | 1428.5 | 1436 | 1297.5 | 1250.5 | 674 | 600 | 569.5 | 704.5 | 550 |
| P-value | .136 | .107 | .037* | .248 | .255 | .927 | .371 | .297 | .646 | .374 |

Note. * indicates p<0.05. Radiation subtype = photon beam versus proton beam radiation.

Mann-Whitney U-tests (RCIs predicted by tumor subtype)

|  | COWA 6m | HVLT-R imm 6m | HVLT-R del 6m | TMT A 6m | TMT B 6m | COWA 1y | HVLT-R imm 1y | HVLT-R del 1y | TMT A 1y | TMT B 1y |
| --- | --- | --- | --- | --- | --- | --- | --- | --- | --- | --- |
| U-value | 1207.5 | 1428.5 | 1436 | 1297.5 | 1250.5 | 748 | 672.5 | 1034 | 517 | 503 |
| P-value | .847 | 0.107 | 0.037* | 0.253 | 0.060 | 0.464 | 0.939 | 0.361 | 0.107 | 0.163 |

Note. * indicates p<0.05. Tumor subtype = defined as glioma versus non-glioma.

Pearson correlations (between RCIs, peak dose, tumor volume and age)

|  | COWA 6m | HVLT-R imm 6m | HVLT-R del 6m | TMT A 6m | TMT B 6m | COWA 1y | HVLT-R imm 1y | HVLT-R del 1y | TMT A 1y | TMT B 1y |
| --- | --- | --- | --- | --- | --- | --- | --- | --- | --- | --- |
| Peak RT dose | -,124 | -,063 | -,007 | -,030 | -,007 | ,159 | ,181 | ,075 | -,077 | -,090 |
| Tumor volume | -,015 | -,264** | -,158 | -,150 | -,009 | ,022 | -,215 | -,318** | -,178 | -,147 |
| Age | -,106 | -,193 | -,108 | -,037 | -,009 | -,001 | ,056 | ,121 | ,052 | ,012 |

Note.** indicates p<0.01.

Table 4. Multiple linear regression analysis models predicting HVLT-R RCIs including confounding factors

Multiple linear regression predicting RCI in HVLT immediate recall at 6m

| **Predictor** | **Estimate** | **Std. Error** | **t value** | **Pr(>\|t\|)** |
| --- | --- | --- | --- | --- |
| (Intercept) | 0.2726 | 0.5450 | 0.500 | 0.6181 |
| RTdose to HVLT imm 6m | -0.06152 | 0.02525 | -2.436 | 0.0167 * |
| Radiation Type | 0.5188 | 0.4433 | 1.170 | 0.2448 |
| Tumor Volume | -0.0000069 | 0.0000045 | -1.521 | 0.1316 |
| Glioma vs non-glioma | -0.2109 | 0.4869 | -0.433 | 0.6659 |

Multiple linear regression predicting RCI in HVLT immediate recall at 1y

| **Predictor** | **Estimate** | **Std. Error** | **t value** | **Pr(>\|t\|)** |
| --- | --- | --- | --- | --- |
| (Intercept) | 1,60E+03 | 0.5247 | 3.056 | 0.00319 ** |
| RTdose HVLT imm 1y | -0.05130 | 0.01887 | -2.719 | 0.00828 ** |
| Radiation Type | -0.4213 | 0.4323 | -0.974 | 0.33325 |
| Tumor Volume | -0.000001056 | 0.000004602 | -0.230 | 0.81911 |
| Glioma vs non-glioma | -1,23E+03 | 0.4963 | -2.474 | 0.01583 * |

Multiple linear regression predicting RCI in HVLT delayed recall at 6m

| **Predictor** | **Estimate** | **Std. Error** | **t value** | **Pr(>\|t\|)** |
| --- | --- | --- | --- | --- |
| (Intercept) | 1,12E+03 | 0.5709 | 1.969 | 0.051900 |
| RTdose HVLT delayed 6m | -0.09314 | 0.02661 | -3.500 | 0.000715 *** |
| Radiation Type | 0.6309 | 0.5057 | 1.248 | 0.215313 |
| Tumor Volume | -0.00000835 | 0.000004907 | -1.702 | 0.092140 |
| Glioma vs non-glioma | -0.9471 | 0.5226 | -1.812 | 0.073145 |

Multiple linear regression predicting RCI in HVLT delayed recall at 1y

| **Predictor** | **Estimate** | **Std. Error** | **t value** | **Pr(>\|t\|)** |
| --- | --- | --- | --- | --- |
| (Intercept) | 2,29E+03 | 0.6126 | 3.736 | 0.000385 *** |
| RTdose HVLT delayed 1y | -0.1074 | 0.02968 | -3.619 | 0.000564 *** |
| Radiation Type | 0.08678 | 0.5203 | 0.167 | 0.868019 |
| Tumor Volume | -0.00001226 | 0.000004777 | -2.566 | 0.012490 * |
| Glioma vs non-glioma | -1,50E+03 | 0.5685 | -2.631 | 0.010535 * |

Note. ‘***’=p<0.001; ‘**’= p<0.01; ‘*’= p<0.05

**References**

1. Sleurs C, Zegers CML, Compter I, et al. Neurocognition in adults with intracranial tumors: does location really matter? *J Neurooncol*. 2022;160(3):619-629. doi:10.1007/s11060-022-04181-7

2. openREGGUI. https://openreggui.org/.

3. Avants BB, Tustison N, Song G. Advanced Normalization Tools ( ANTS ). Published online 2011:1-35.

4. Benton LA, Hamsher KD, Sivan AB. Controlled oral word association test, multilingual aphasia examination. *AJA Associate*. Published online 1994.

5. Siciliano M, Chiorri C, Battini V, et al. Regression-based normative data and equivalent scores for Trail Making Test (TMT): an updated Italian normative study. *Neurological Sciences*. 2019;40(3). doi:10.1007/s10072-018-3673-y

6. Benedict RHB, Schretlen D, Groninger L, Brandt J. Hopkins verbal learning test - Revised: Normative data and analysis of inter-form and test-retest reliability. *Clinical Neuropsychologist*. 1998;12(1). doi:10.1076/clin.12.1.43.1726
